# Beginning community engagement at a busy biomedical research programme: Experiences from the KEMRI CGMRC-Wellcome Trust Research Programme, Kilifi, Kenya

**DOI:** 10.1016/j.socscimed.2008.02.007

**Published:** 2008-09

**Authors:** Vicki Marsh, Dorcas Kamuya, Yvonne Rowa, Caroline Gikonyo, Sassy Molyneux

**Affiliations:** a KEMRI Wellcome Trust Research Programme, Kilifi, Kenya; b Oxfam, Kenya

**Keywords:** Kenya, Community engagement, Bioethics, Ethical research

## Abstract

There is wide acknowledgement of the need for community engagement in biomedical research, particularly in international settings. Recent debates have described theoretical approaches to identifying situations where this is most critical and potential mechanisms to achieve it. However, there is relatively little published experience of community engagement in practice. A major component of the Kenya Medical Research Institute (KEMRI) Wellcome Trust Research Programme is centred on Kilifi District General Hospital and surrounding community of 240,000 local residents. Documented community perceptions of the research centre are generally positive, but many indicate a low understanding of research and therapeutic misconceptions of its activities. As in other settings, these misunderstandings have contributed to concerns and rumours, and potentially undermine ethical aspects of research and local trust in the institution. Through a series of consultative activities, a community engagement strategy has been developed in Kilifi to strengthen mutual understanding between community members and the Centre. One important component is the establishment of a representative local resident network in different geographic locations commonly involved in research, to supplement existing communication channels. Early implementation of the strategy has provided new and diverse opportunities for dialogue, interaction and partnership building. Through the complex social interactions inherent in the community engagement strategy, the centre aims to build context specific ethical relations with local residents and to strengthen understanding of how ethical principles can be applied in practice. Evaluations over time will assess the effectiveness and sustainability of these strategies, provide generalisable information for similar research settings, and contribute to debates on the universality of ethical principles for research. This paper aims to summarise the rationale for community engagement in research, drawing on published literature and local findings, to outline the process of community engagement in Kilifi and to describe issues emerging from its development and early implementation.

## Introduction

Increasing recognition of the need to consider the ethical implications of biomedical research participants as members of a wider community, and not just as individuals, has led to active international debate on the value, goals and practicalities of involving communities in many aspects of the planning and conduct of research. (Dickert & Sugarman, 2005; Emanuel, Wendler, Killen, & Grady, 2004; Gollust, Apse, Fuller, Miller, & Biesecker, 2005; Newman, 2006; Quinn, 2004; Strauss et al., 2001; Weijer, Goldsand, & Emanuel, 1999; Weijer & Miller, 2004). Although key ethical principles for biomedical research are well recognised, the primary rationale for community engagement is premised on widely acknowledged challenges in contextualising and applying these principles in different research environments (Belmont, 1979; CIOMS, 2002; Emanuel et al., 2004; Nuffield Council on Bioethics, 2002; Quinn, 2004).

Community engagement is highly pertinent in our setting, a busy multidisciplinary long-term biomedical research institute, with significant international donor support, set in a district general hospital in a poor rural area in Kenya (KEMRI, 2006). Documented community perceptions of the research centre are generally positive, but many describe a low understanding of research and therapeutic misconceptions of its activities (Molyneux, Peshu, & Marsh, 2004; Molyneux, Wassenaar, Peshu, & Marsh, 2005). As in other settings (Geissler & Pool, 2006; Leach & Fairhead, 2006; Mitchell, Nakamanya, Kamali, & Whitworth, 2002). These misunderstandings have contributed to concerns and rumours, and potentially undermine ethical aspects of research and local trust in the institution (Molyneux, Peshu, & Marsh, 2005).

Through a series of consultative activities, a community engagement strategy has been developed in Kilifi to strengthen mutual understanding between community members and the research centre. One important component is the establishment of a representative local resident network in different geographic locations commonly involved in research, to supplement existing channels of communication. This paper aims to summarise the rationale for community engagement in research, drawing on published literature and local findings, to outline the process of community engagement in Kilifi and to describe issues emerging from its development and early implementation.

## Rationale and goals for community engagement

There is wide agreement on the importance of community engagement in many areas of research and types of research settings. Most pressure for, and experience with, community involvement in biomedical research has come from studies on aboriginal communities, HIV/AIDS, emergency medicine, international research and, more recently, genetic diversity (AIATSIS, 2002; Emanuel et al., 2004; HGDP, 1999; Morin, Maiorana, Koester, Sheon, & Richards, 2003; Weijer & Miller, 2004). Authors point to four main goals for community involvement: protection, respect, empowerment and partnership building (Dickert & Sugarman, 2005; Foster et al., 1999; Lavery, 2004; Marshall & Rotimi, 2001; Morin et al., 2003; Quinn, 2004; Sharp & Foster, 2000; Weijer & Miller, 2004).

Involving communities in planning and conducting research is a means of identifying and minimising internal risks (those only visible within a community), such as social identity and equilibrium (Sharp & Foster, 2000). Including local viewpoints can also minimise external risks to the community, such as stigmatisation and its potential economic, psychosocial and health consequences. In addition to addressing community interests, representatives can strengthen individual protection in research by supporting informed consent processes through dissemination of information on research goals, risks and benefits and incorporating local views into the development of informational aspects of research (Strauss et al., 2001). According communities rights to comment on the planning and conduct of research affecting them is empowering; it demonstrates respect, provides opportunities for maximising benefits for communities and increases mutual understanding (Lavery, 2004). Greater mutual understanding may strengthen research processes. It may also increase community uptake of any products of that research (Dietrich & Schibeci, 2003; Sharp & Foster, 2000). Increasing awareness of other viewpoints may help individuals to subordinate their preferences or differences to benefit a larger community (Macpherson, 2004). Some authors have pointed to a practical benefit of collaborative processes to researchers of greater credibility (Parkin, 2004). For some forms of research, such as participatory action research, community involvement is a defining characteristic, shown by shared goals, decision-making and benefits for researchers and communities (Macaulay et al., 1999). Overall, Quinn describes the value of a “relationships paradigm” for research ethics, where researchers are able to anticipate and address the context in which communities understand risks and benefits, and individuals give consent. This process of giving a voice to communities involved in research is advocated in place of traditional ethics models based on the application of universal principles.

It is reasonably argued that community involvement may not be appropriate in all types of and settings for research (Weijer & Emanuel, 2000), but the relevance of community involvement has been increasingly articulated for international research (Diallo et al., 2005; Doumbo, 2005; Emanuel et al., 2004; Weijer & Miller, 2004). The importance of affording greater protection, respect and empowerment to communities that participate in international research is underlined by the differences in social and cultural norms, values, goals, resources and technological understanding between researchers and typical participant communities (Doumbo, 2005; Leach & Fairhead, 2006; Mitchell et al., 2002; Molyneux, Wassenaar, et al., 2005). Weijer stresses the importance of empowerment as an ethical requirement in research involving vulnerable, oppressed and non-majority groups (Weijer & Miller, 2004). Lavery (2004) points to the need for consultation and negotiation to ensure more equitable distribution of the benefits of research in low and middle income countries. Community engagement may provide a mechanism for retaining a shared institutional memory for researcher–community interactions over time. This is arguably of particular importance in long-term international research centres, where formal governance may provide less representation, and therefore protection, to individuals.

## Types of community involvement in research

Various forms of community involvement have been described, with a range of expressed goals. A key point of difference is the balance of power between researchers and participant communities. Sharp describes a spectrum of power sharing, from community dialogue through community consultation and approval to full partnership, where the latter implies greatest community empowerment (Sharp & Foster, 2000). Similarly, a summary of public participation techniques by the New Economic Foundation in the United Kingdom describes a hierarchy of methods ranging from non-participatory techniques of manipulation, through “tokenistic” approaches of placation, informing and consultation, to power sharing strategies of partnership and delegated or citizen power (New Economic Foundation, 1999). Using this model, community engagement mechanisms towards the bottom of this “ladder of participation” could include information dissemination about planned research, while activities at the top might provide legally constituted representative community groups powers of veto in relation to proposed research.

Weijer and Sharp's analysis that potential forms of engagement are predicated on specific community characteristics provides a helpful conceptual framework (Sharp & Foster, 2000; Weijer, 2004; Weijer & Emanuel, 2000). “Consultation with consent” is only achievable in settings where communities have legitimate political authorities. “Consultation” requires authentic community representation, where authenticity implies fair, balanced and accurate representation of the many and varied constituencies within a community. For communities with low degrees of coherence (for example, “communities” defined by disease states), factors such as low internal risk, absent means of representation and poor internal communication render community involvement less relevant. Other authors recognise characteristics of the research (in particular, risk-benefit analyses) to be important factors in gauging the importance of community involvement (Ernst & Fish, 2005). However, there is wide consensus that community representatives can potentially be involved in a broad range of activities in research, from protocol development (including the process of providing information and obtaining consent), to research conduct, reviewing access to data and samples, and dissemination or publication of research findings (Marshall & Rotimi, 2001; Quinn, 2004; Sharp & Foster, 2000; Weijer & Miller, 2004).

Probably the most prominent mechanism for community engagement in international research has been the use of Community Advisory Boards (CAB), defined as “being composed of committee members who share a common identity, history, symbols and language, and culture” (p. 1940) (Strauss et al., 2001). Marshall describes CABs as an example of a strategy “safeguarding the interests of local populations, through the establishment of a solid foundation that supports a relationship based on trust and engagement with community members” (p. 243) (Marshall & Rotimi, 2001). Of key importance is the establishment of a relationship that is sustained over time, specifically beyond the lifetime of any specific research project. However, a major challenge to the CAB model has been identifying stakeholders with legitimate interests – that is, avoiding politicisation and ensuring authentic community representation (Dickert & Sugarman, 2005; Foster et al., 1999; Marshall & Rotimi, 2001; Mills et al., 2005; Sharp & Foster, 2000). Underpinning these difficulties are debates on how to define the community (Ernst & Fish, 2005; UNAIDS, 2006), balancing remuneration and independence of members (Morin et al., 2003), the need for resources to train and sustain CAB activities, and resolving differences between community-level and individual decisions (Quinn, 2004; Sharp & Foster, 2000). Given the importance of lead researchers in negotiating solutions to many of these potential challenges, the effectiveness of the CAB has been described as being determined by the relationship between a principal investigator and the community (Sharp & Foster, 2000). Examples of politically powerful community representatives have emerged in HIV/AIDS research, highlighted recently in three studies on HIV prophylaxis in Cameroon, Cambodia and Nigeria where the actions of activists representing community interests led to a cancellation of research in planning or in progress (Mills et al., 2005). The authors describe that “the issues raised by activists, academics and the research community highlight the poor communication between these stakeholders and the need for mutual understanding of values” (p. 1403).

## Research gaps for community engagement

Given the paucity of published experience and empirical data on community engagement, many authors have acknowledged the need for further research, including the identification of authentic community representatives, methods of engagement and situations when engagement is needed (Diallo et al., 2005; Foster, Eisenbraun, & Carter, 1997; Morin et al., 2003). Guidelines exist for community–researcher interactions in some specific settings (for example, AIATSIS, 2002; NBAC, 1999), and there have been repeated calls for guidance on the development and implementation of community engagement processes for a broader range of situations (Mitchell et al., 2002; UNAIDS, 2006; Weijer, Goldsand, & Emanuel, 1999). There are also many unanswered questions about the way that representative community groups or individuals do or should function, such as how they interact with Institutional Review Boards (IRBs), what role they play in developing informed consent processes and supporting informational activities, whether they act to empower historically vulnerable groups and how the balance of power between researchers and community representatives is negotiated (Diallo et al., 2005; Dickert & Sugarman, 2005; Quinn, 2004; Sharp & Foster, 2002). A specific need has been identified for more carefully designed studies on the impact of such collaborative efforts on research design and implementation, to understand how representative community groups work to guide, speak for and protect their communities (Sharp & Foster, 2000). Given that current ethical principles are founded on a notion of equity between researchers and participants, the impact of community engagement on this relationship is of fundamental ethical importance.

## The research centre and participant communities in Kilifi

**Fig. 1:**
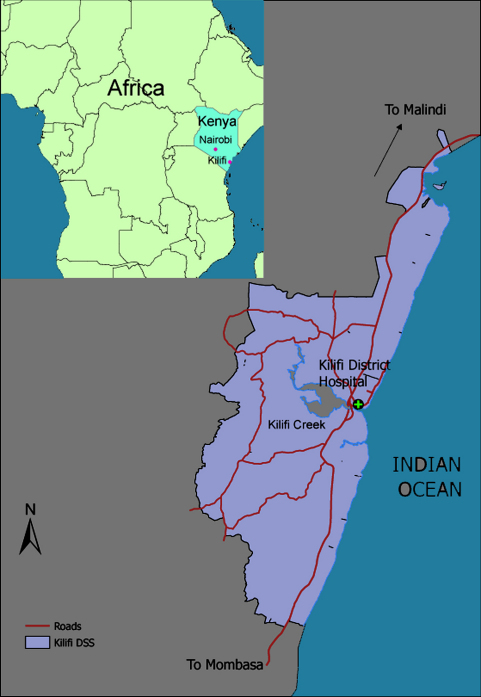
Kenya, Kilifi district and the research study area (the DSS area).

Kilifi district lies on the coast of Kenya (see Fig. 1), with a projected population of approximately 653,144 for 2006 (CBS, 2002). The residents are primarily from the Mijikenda ethnic group. The economy is based on subsistence farming with some support from low-level tourism and trade in Kilifi town and nearby larger urban centres. The main religious denominations are Christianity and Islam, with approximately equal proportions of these represented. Traditional religions are also followed, particularly in inland rural areas. The district has high level of poverty; although the DSS area contains both less (largely urban) and more (mainly rural) poor residents, poverty incidence rates for the two political constituencies contained within the Demographic Surveillance System (DSS) area range from 65 to 84%, including the highest rates in the country (CBS/GOK/WB/SIDA/SID, 2005). Literacy rates are low; in 329 randomly surveyed households in the DSS area in 2005, 45% of adults reported being able to read a newspaper or letter.

The Kenya Medical Research Institute (KEMRI) Centre for Geographical Medicine Research, Coast (CGMRC) is one of 10 research centres in Kenya administered by KEMRI, a parastatal organisation under the Ministry of Health. A collaborative research programme was set up in Kilifi between KEMRI CGMRC and the Wellcome Trust in 1989, and currently attracts support from several international funding agencies. The centre has developed a strong international reputation for its wide-ranging interdisciplinary research covering clinical, basic science, epidemiological and public heath aspects of major childhood and adult diseases, focused primarily on concerns for coastal Kenya. A key feature of the programme has been its deliberate development within a District Hospital, with research being carried out in a “real world environment” serving a rural community. The research centre provides support to the hospital to ensure a good standard of care is available to those using the departments where research is conducted, regardless of their involvement in research. The additional resources include medical and clinical officers, paediatric drugs and equipment and a paediatric intensive care ward. Within the community, clinical services are supported at specific government health centres and dispensaries. A Demographic Surveillance System (DSS) has been established in the area surrounding the hospital. Approximately 240,000 people are included, accounting for around 80% of all paediatric hospital admissions. Homes within the DSS are visited three times a year to collect information on residence, migration, births and deaths. A map showing the hospital and DSS area is shown in Fig. 1. The centre is represented in the District Development Committee, a coordinating group for all district government departments, such as health, education and social services.

By 2001, parents were signing consent for over 4000 children per year to be involved in clinical studies at the research centre ranging from purely observational research to the testing of new procedures and drugs. Thousands more community members were consenting verbally or in writing to interviews and procedures in community-based research. Every study carried out by the programme has always been scrutinised in advance by local and independent national and international scientific and ethical review committees. The existing methods of community engagement included consultation with local administrative leaders (chiefs) in advance of all community-based research, dissemination of information through these leaders and through public meetings, interpersonal communication between staff and the community and use of print materials.

## Developing a community engagement strategy

**Fig. 2:**
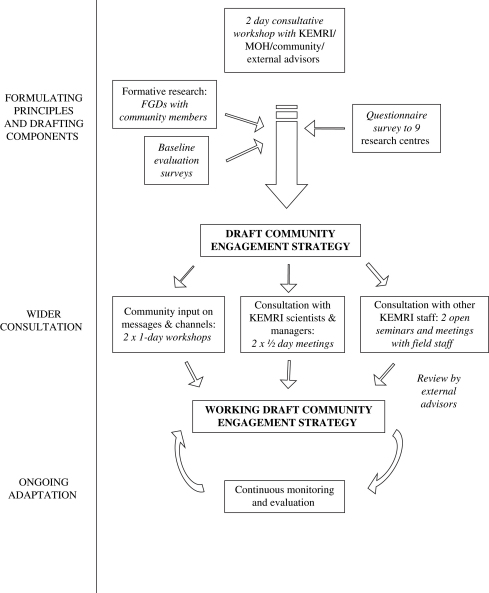
Steps to developing a community engagement strategy.

In response to increasing recognition of community misconceptions about research and their ethical implications (Molyneux, Peshu, & Marsh, 2004, 2005; Molyneux, Wassenaar, et al., 2005), and to the community engagement issues raised in the international literature, specific funds were obtained to develop and implement a communication strategy for the centre. This had three main goals: strengthening partnership with key stakeholders; promoting adherence with good clinical practice and ethical guidelines for research; and ensuring long-term sustainability of the programme. Community members from the DSS area were identified as key stakeholders and strategic steps outlined for community engagement to strengthen communication and interactivity, and build greater mutual understanding. This paper focuses primarily on the community engagement components of the communication strategy. More information on the process of developing the overall communication strategy will be provided in a separate publication. In summary, the main steps were (see Fig. 2):

• Further formative research on community perceptions of the research centre to establish homogeneity across the DSS area and seek community views on engagement processes;
• Input from an external advisory group (five experts in ethics, health policy and communication) on strategic planning for the overall communication strategy, including community engagement;
• A 2-day consultative workshop for external advisors, community representatives, a member of the District Health Management Team (DHMT) and KEMRI researchers to develop a draft communication strategy;
• Wide consultation on the draft communication strategy with centre staff, community members and the DHMT to develop the working draft that currently provides a guide for implementation of the community engagement strategies outlined in this paper.

### Achieving community representation

Two challenges to establishing community representation were defining which community, or grouping within a community, should be represented, and establishing a mechanism by which fair, balanced and accurate representation could be achieved. As has been frequently recognised, “community” is a widely used and highly flexible term. A definition that encapsulates the breadth of the term as commonly used is “a group of people sharing a common interest – for example, cultural, social, political, health and economic interests, but not necessarily a particular geographic association” (Brown & Tandon, 1983). Within the centre's communication strategy, the community was defined as the normal residents of the DSS area, but mechanisms for effecting authentic representation of this heterogeneous population were not clear.

Civil administration in Kenya is conducted through the Office of the President, a government department with tiers at national, provincial, district, divisional, locational and sublocational levels. The DSS area covers, approximately, half the district, including 3 divisions divided into 14 locations. Amongst these, project specific CABs had been established in the past in four locations, with members selected by chiefs. Further potential channels for engagement within these locations included local councillors, Village Development Committees (VDCs) and community-led Dispensary Health Committees (DHCs) associated with rural health facilities. Each presented challenges to community representation either through their own selection processes or through non-uniform geographic coverage and potential under-representation of certain constituencies (women, young people and the most rural). Discussions with key informants in government social services and health departments as well as the researchers' local knowledge of this community indicated that the wide network of community-based organisations (CBOs) active across the study area could potentially provide authentic representation. Some CBOs were registered with the social services department, but documentation of the types, activities and membership was hindered by a lack of resources for monitoring.

**Fig. 3:**
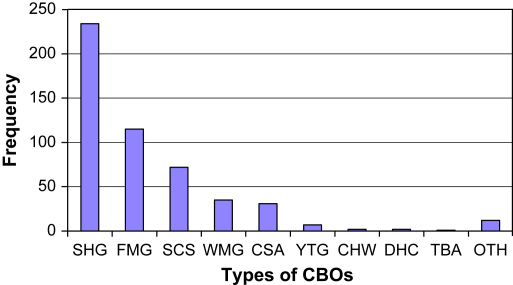
Types of Community-based organisations in 10 locations in Kilifi district. SHG = self help group; FMG = farmers group; SCS = social or cultural group; WMG = women's group; CSA = credit and savings group; YTG = youth group; CHW = community health workers; DHC = dispensary or health centre committee; TBA = traditional birth attendant; and OTH = other.

We conducted a survey of all active CBOs in 10 DSS locations without a CAB, using the social services database and a snowballing technique involving chiefs, assistant chiefs and CBO members. At least two members of each CBO were interviewed together, using a semi-structured questionnaire to gather information on the membership and functioning of the group. We identified 569 groups, one-third of which were unregistered, with a median active membership of 16 people (Inter Quartile Range10–22) across an area with a population 98,117, giving a ratio of 1 active CBO member to, approximately, 11 people in the community. This ratio, and the range of membership and activities (shown in Fig. 3), supported our proposal that this channel might provide balanced and accurate community representation. A further advantage was that it would supplement, and not duplicate, existing channels of communication with higher profile community groups, such as the chief's office, VDCs and DHCs. The CBO network was therefore chosen as the basis for identifying representative community members in these 10 locations, as described below.

### Beginning community engagement

In the locations with an inactive or no CAB, CBO representatives nominated between 10 and 19 individuals from active groups to represent each location, depending on the population numbers and density. The role of the community representatives was introduced as a voluntary undertaking to strengthen communication between KEMRI and the community. Representatives' responsibilities included participation in regular quarterly community-based meetings, and *ad hoc* communication when needed, with KEMRI liaison staff. Travel expenses would be reimbursed for quarterly meetings. Communication with local residents would be informal interactions with other community members as part of their normal family life and CBO activities. Nominees were selected by consensus at a series of meetings of CBO representatives at the research centre. Nominees, chiefs and other community gatekeepers later attended one of five 2-day participatory workshops on KEMRI, health research and the rights of participants in research. At these workshops, they discussed representatives' roles and selected the name of KEMRI community representatives (KCRs) for the network. Following training, chiefs organised a series of large scale public meetings in each location, facilitated by the research centre and the Ministry of Health, to seek community endorsement for the nominated individuals, and disseminate information on the KCR's roles. Nominees from the CBO selection process were all endorsed as KCRs. In three locations where pre-existing active CABs had precluded nominations by CBO representatives, the individuals were not endorsed. The main reasons for their rejection were their perceived lack of geographic representation or political bias. Chiefs in these locations countered that difficulties in finding volunteers had led to uneven geographic coverage. Later consultation with wider leadership groups representing all geographic areas of these locations led to the nomination and later public endorsement of new KCRs. Since endorsement, quarterly and *ad hoc* community-based meetings between KCRs and KEMRI liaison staff have begun to strengthen existing communication channels. Through this linkage, there has been greater feedback on community concerns and recommendations to KEMRI (including providing advice on specific aspects of research planning), and dissemination of information on different aspects of KEMRI's work in the community.

### Emerging issues

#### The value of qualitative formative and action research

The value of rigorous qualitative research in developing a community engagement strategy (Newman, 2006) is strongly borne out by our experiences. Qualitative studies in 2001 and 2004 brought out clearly the mix of communication, environmental and institutional policy issues, including power dynamics, forming the backdrop to KEMRI-community relations (Molyneux et al., 2004). It provided an explanation of the source of commonly encountered community concerns and rumours as rational attempts to fill a gap in understanding, using a mixture of past experiences, traditional beliefs and fragments of religion and folklore. Thus, for example, we learned that concerns about the snake depicted in both the KEMRI and, at that time, the Wellcome Trust's logos arose from a common belief that snakes are a symbol of devil worshipping. Further, a local belief held that encountering two snakes intertwined (as shown in the old Wellcome Trust logo) foretold a death in the family. Rumours of devil worship were strengthened by the prominence of blood taking as a research activity in the absence of an understanding of research, as has been reported elsewhere (Leach & Fairhead, 2006; Mitchell et al., 2002). Two important examples of institutional policy issues drawn out through formative research were the community's concerns about KEMRI's employment policies and the training needs of field workers and other staff at the interface of KEMRI-community interaction.

Action research methods have been used throughout the development and implementation of the community engagement strategy, described as ‘a (usually cyclic) process by which change and understanding can be pursued at the one time, with action and critical reflection taking place in turn. The reflection is used to review the previous action and plan the next one’. In the latter cycles of action research, methods, data and interpretation are continually reshaped in the light of the understanding developed in the earlier cycles. Involvement of the community in action research to identify representatives, activities, channels and messages has strengthened the potential effectiveness of these components and has been a critical step in demonstrating the centre's commitment to community involvement. Building on a wide consultative process, research staff perspectives of the community engagement strategy have evolved towards seeing greater community involvement as a fundamental way in which the centre could strengthen certain ethical aspects of research, as described elsewhere (Emanuel et al., 2004).

#### The role of an external advisory group

The external advisory group provided technical support, objectivity and perspectives on generalisability in developing the overall communication, and community engagement, strategies. There is an obvious risk of bias (inadvertent or otherwise) in using an internal process to develop strategies to strengthen ethical aspects of research. Although a protocol for this work was routinely reviewed and approved by local, national and international scientific and ethical review bodies, the nature of action research implies that continuing objective, technical advice is an important part of this overview. The technical expertise of the external advisory group brought an increased depth of understanding of the issues behind KEMRI-community relations and greater coherence to the strategies developed to address them. The group were important in advocating a move away from early concepts of simple communication activities, such as the production and dissemination of print materials, towards a broader communication strategy cross-cutting all departments, including policy.

#### Resources and flexibility

A variety of activities underpinned the development and implementation of the community engagement strategy. The resources needed were significant (Newman, 2006) and often unpredictable, including time, personnel, skills (communication, facilitation, participatory training, negotiation) and funding. Setting up and maintaining a network of KCRs has entailed surveying existing community structures, outreach activities, nominations and public endorsements, participatory trainings and regular meetings. Adequate human resources (Lavery, 2004), such as time and skills, and flexibility were major challenges for a biomedical research centre where the highly competitive nature of funding tends to limit amounts allocated to non-research elements and tie activities to an agreed time line. On the other hand, within a collaborative framework, the community was able to contribute resources for many activities, such as their time in planning and advertising public meetings and provision of venues and equipment for these.

#### Assessing the effectiveness of community engagement

Given the complexity of the goals and mechanisms, it is not surprising that community engagement initiatives are difficult to evaluate (Dickert & Sugarman, 2005). Equally, given these complexities and the resources required, it is particularly important to understand effectiveness in ways that justify the investment and inform the process. In Kilifi, we are using a combination of quantitative and qualitative methods to assess processes and impacts over time. In the baseline survey, we found important challenges in using quantitative methods to evaluate understanding, given the ambiguities of local language around research and treatment. It was not possible, even after prolonged pre-testing, to develop structured questionnaires to measure understanding of research. Some level of open discussion was always needed to ascertain meaning. Our final tool was a semi-structured questionnaire that was coded at the end of the interview on the basis of responses occurring in any part of the questionnaire. It is possible that one measure of success for engagement will be that quantitative tools become easier to use in future surveys but qualitative methods will remain key to understanding and validating quantitative findings. Extensive pre-testing of tools, qualitative skills for interviewers, and a detailed manual to ensure that, as far as possible, surveys can be replicated in a comparable way in the future, are important components to such an evaluation.

#### Generalisability and representativeness

The research setting in Kilifi provides a specific, though not unique, example of researcher–community interactions defined by the presence of a busy, long-term and relatively well resourced international biomedical research centre in a geographically fixed and relatively poor rural population in sub-Saharan Africa. The mechanisms developed here for community engagement may be either less necessary or inappropriate in other settings, such as research centres within tertiary level health facilities in urban settings. Strategic approaches to developing appropriate levels and types of community engagement have been described (Weijer & Miller, 2004) based on the characteristics and structure of research communities, and the type of research. Important community characteristics are described as: common culture and traditions; knowledge and shared history; comprehensiveness of culture; health-related common culture; legitimate political authority; representative groups or individuals; mechanism for priority setting; geographic localization; common economy and shared resources; communication network; and self identity as a community. For example, community consultation requires authentic representative voices and effective communication within the community to underpin current and continuing authenticity. This may be achievable in cohesive communities through existing social units (Sharp & Foster, 2002). In the absence of authentic representation and a communication network, Weijer suggests that less formal types of dialogue could replace community consultation, which may be neither achievable nor needed. However, he notes that certain types of community – specifically those that are vulnerable, oppressed or minority groups – may require formal consultation with legitimate representatives without existing mechanisms for achieving this. This latter situation pertains in Kilifi, and may be typical of many international research settings, given the vulnerability implied by poverty, low access to education and unmet health needs. Our challenge, then, was to identify how representative community voices could be brought into discussions on research planning and debates on research ethics and governance.

Our approach to setting up a long-term representative mechanism for community engagement in Kilifi has drawn on the existing CBOs that operate across a broad range of activities within this community and therefore seem likely to both represent and interact with the majority of constituencies. As a result of the mechanism for its formation, the KCR network draws from existing groups within the community, with important implications for sustainability as well as representation. We have evidence from the CBO survey that these groupings provide close links to a wide sector within this community. There is also evidence from the endorsement process that the CBO approach is more acceptable to community members attending public meetings than representatives identified by chiefs, reflecting wider concerns about “how authentically people appointed by agencies outside a group speak for the group” (CIOMS, 1991). The finding highlights the importance of public endorsement as a check for fairness, accuracy and balance of individuals chosen to represent them. At the same time, we are cautious of the extent to which a CBO network may function to achieve this representation, and communicate with the wider community over time. We are also observant of the need to ensure that the KCR network supplements existing channels for communication with local residents, such as chiefs, KEMRI and MOH staff, rather than replace them. A single strong channel may both lose authenticity and risk over-politicisation.

#### Ownership and partnership

A corollary of the process of developing community representation mechanisms *de novo* is that early stages were inevitably research centre led. Thus, while KEMRI aimed to facilitate participatory processes to underpin development of a community engagement strategy, community members perceived the research centre to have primary ownership and responsibility for setting up and maintaining the KCR structure. Given the low understanding of research in this community, comparisons were made with local non-governmental community development organisations where the benefits of mutual cooperation were clear. The relative wealth of the centre, seen through resources such as four-wheel drive vehicles, medical supplies and well-equipped buildings, initially led to demands for KEMRI to support KCRs through the provision of offices, payments, telephones and transport. Such demands would potentially threaten wider community perceptions about the independence of KCRs, and call into question which “community” they represent (Morin et al., 2003). Although these demands have reduced with greater understanding of research and the KCR role, the challenge of providing sufficient resources for KCRs to be effective while maintaining their perceived independence from the centre remains. The relative wealth of the research centre forms a continuing backdrop to these negotiations. To counterbalance inequities, policies are being developed within the research centre to strengthen inputs to local health service provision, through bilateral discussions on MOH–KEMRI interactivity.

Community engagement is a two-way process, and the attitudes of research staff a critical element. A principal investigator who is willing to listen and act on feedback where necessary is a requirement for effective engagement (Sharp & Foster, 2000; Strauss et al., 2001). In Kilifi, research and administrative staff have expressed wide support for community engagement. Researchers' main aims were to respect community views, respond to community concerns (particularly those impacting negatively on recruitment) and provide systematic community feedback of findings. For specific projects, such as long-term cohort studies on genetics and disease, there was a perceived need for consultation on protocol development. Reservations were voiced about community engagement in developing research agendas, and the potential for complex explanations on highly technical areas of research to cloud fundamental ethical issues of autonomy. A second key staff group were field workers, whose primary responsibilities are conducting interviews, and sometimes collecting samples, from research participants. They are recruited from the local community and represent KEMRI during their daily work, undertaking a critical role as cultural brokers (Molyneux, Peshu, et al., 2005). Discussions with field workers and community members highlighted issues with important implications for interactions between them. Important examples were field workers' perceived need to recruit adequate numbers of research participants, their empathy with participants' interest in individual benefits, and their low awareness of local and international guidelines for ethical research conduct. Routine field worker training on research and communication has subsequently been strengthened and expanded to include research methods and participants' rights.

There are evident complexities in developing systems to coordinate interactivity and partnership building between a variety of both community and research constituencies that will answer concerns and needs of all (for example, to enable staff from a range of different individual projects and KCRs to respond effectively to local issues and concerns, and to facilitate research). An example of the complexity of this process is described for a specific Malaria Vaccine Trial at the research centre in this issue (Gikonyo, Bejon, Marsh, & Molyneux, 2008). However, even at this early stage, the KCR network has led to changes in institutional policy with apparent direct benefits to the community and some costs to the research centre. Feedback from the community has led to changes in employment policy, with all non-scientific posts now being advertised within the community and the development of specific written recruitment guidelines. The centre's vehicles now carry only institutional names, not logos. All new research proposals must consider the need and mechanisms for community engagement. Facilities at the centre, such as seminar rooms, have been opened up for community use. As described, resources have been made available to strengthen training of field workers who regularly interact with the community. Issues raised by KCRs from the community at regular quarterly meetings, ranging from requests for information and recommendations on specific studies to complaints about staff conduct, have been communicated to researchers and acted upon. At the same time, community members have contributed essential resources to the community engagement process, such as their time and local planning support for activities. For example, in some instances KCRs have accompanied KEMRI liaison staff in visiting homes to respond to serious concerns or complaints from community members. Beyond the practical value that these community contributions represent, they may also chart an early shift for ownership of the community engagement process away from the research centre and towards the community, with implications for the balance of power between these parties.

Since partnership models of engagement are based on mutual understanding and shared decision-making and benefits, these examples of changes in institutional policy to benefit the community and community contributions to benefit the research centre may plausibly be described as reflecting a partnership. However, researchers perceived research review processes as less easy to adapt to this level of power sharing, a view acknowledged by other authors (Marshall & Rotimi, 2001; Morin et al., 2003; Quinn, 2004; Sharp & Foster, 2000; Weijer, 2004). A consultative model (that is, listening to and incorporating community perspectives) may offer a more effective strategy for research review, and is the approach currently followed in Kilifi. In this situation, inputs from broader groups of stakeholders representing community views have also been essential. Particularly key groups have been chiefs and MOH staff, illustrating the mutually supportive and often interdependent roles of these channels. Our use of both partnership and consultative models in community engagement illustrates the subtlety of the concept of power sharing. Community engagement in Kilifi has involved complex interactions and negotiations leading to a mix of outcomes that aim to satisfy the main requirements of involved parties. In future, after planned evaluations of KCR functioning, it may be important to ensure that the views of KCRs are also directly visible to the national ethical review body to ensure that consultation provides fair consideration of community interests (Ernst & Fish, 2005).

## Conclusions

King et al. introduced the concept of a relationships paradigm for research in place of a model based on universal ethical principles, or “principalistic model”. He wrote “The moral principles held to govern research with human subjects remain current and meaningful, but make sense only in context. Thus the ethics of human subjects research may be universal but is at the same time deeply particularized, so that what autonomy or informed consent or even benefit and harm means depends on the circumstances” (p. 921) (Quinn, 2004). The recently proposed additional ethical principle of “respect for communities” and guidelines on when and how to establish mechanisms for community engagement move the debate towards ways of defining goals, identifying characteristics and avoiding pitfalls in understanding a local context for ethical principles. We have drawn on this body of opinion to develop and begin to implement mechanisms for community engagement in a busy biomedical international research centre in Kilifi. Emerging issues in the process of development and early implementation have been described in this paper. Key elements of building trust and mutual understanding with community representatives have been shared ownership of the liaison processes and flexibility in power sharing. Local representatives have considered their inputs into institutional policy to be critical and their recommendations in these areas have been adopted. Community input into research review has emerged as a consultative process, although this may evolve over time with greater community understanding of research. Through the complex social interactions inherent in the current community engagement strategy, the centre aims to build context specific ethical relations with local residents and to strengthen understanding of ways in which ethical principles can be applied in practice. Evaluations over time will assess the effectiveness and sustainability of these strategies, provide generalisable information for similar research settings, and contribute to debates on the universality of ethical principles for research.

## References

[bib1] AIATSIS (2002). Guidelines for ethical research in indigenous studies.

[bib2] Belmont (1979). Ethical principles and guidelines for the protection of human subjects of research.

[bib3] Brown L., Tandon R. (1983). Ideology and political economy in inquiry: action research and participatory research. Journal of Applied Behavioral Science.

[bib4] CBS (2002).

[bib5] CBS/GOK/WB/SIDA/SID (2005). Who and where are the poor? A constituency level profile.

[bib6] CIOMS (1991). International guidelines for ethical review of epidemiological studies. http://www.cioms.ch/frame1991textsofguidelines.htm.

[bib7] CIOMS (2002). International ethical guidelines for biomedical Research involving human subjects. 14983848

[bib8] Diallo D.A., Doumbo O.K., Plowe C.V., Wellems T.E., Emanuel E.J., Hurst S.A. (2005). Community permission for medical research in developing countries. Clin Infect Dis.

[bib9] Dickert N., Sugarman J. (2005). Ethical goals of community consultation in research. American Journal of Public Health.

[bib10] Dietrich H., Schibeci R. (2003). Beyond public perceptions of gene technology: community participation in public policy in Australia. Public Understanding of Science.

[bib11] Doumbo O.K. (2005). Global voices of science. It takes a village: medical research and ethics in Mali. Science.

[bib12] Emanuel E.J., Wendler D., Killen J., Grady C. (2004). What makes clinical research in developing countries ethical? The benchmarks of ethical research. Journal of Infectious Diseases.

[bib13] Ernst A.A., Fish S. (2005). Exception from informed consent: viewpoint of institutional review boards – balancing risks to subjects, community consultation, and future directions. Academic Emergency Medicine.

[bib14] Foster M.W., Eisenbraun A.J., Carter T.H. (1997). Communal discourse as a supplement to informed consent for genetic research. Nature Genetics.

[bib15] Foster M.W., Sharp R.R., Freeman W.L., Chino M., Bernsten D., Carter T.H. (1999). The role of community review in evaluating the risks of human genetic variation research. American Journal of Human Genetics.

[bib16] Geissler P.W., Pool R. (2006). Editorial: popular concerns about medical research projects in sub-Saharan Africa – a critical voice in debates about medical research ethics. Tropical Medicine and International Health.

[bib17] Gikonyo C., Bejon P., Marsh V., Molyneux S. (2008). Taking social relationships seriously: lessons learned from the informed consent practices of a vaccine trial on the Kenyan Coast. Social Science & Medicine.

[bib18] Gollust S.E., Apse K., Fuller B.P., Miller P.S., Biesecker B.B. (2005). Community involvement in developing policies for genetic testing: assessing the interests and experiences of individuals affected by genetic conditions. American Journal of Public Health.

[bib19] HGDP (1999). Model ethical protocol for collecting DNA samples.

[bib20] KEMRI (2006). Kenya Medical Research Institute Wellcome Trust Research Programme. http://www.kemri-wellcome.org/.

[bib21] Lavery J.V. (2004). Putting international research ethics guidelines to work for the benefit of developing countries. Yale Journal of Health Policy, Law and Ethics.

[bib22] Leach, M., & Fairhead, J. (2006). Being “with MRC”: Infant care and the social meanings of cohort membership in Gambia's plural therapeutic landscapes.

[bib23] Macaulay A.C., Commanda L.E., Freeman W.L., Gibson N., McCabe M.L., Robbins C.M. (1999). Participatory research maximises community and lay involvement. North American Primary Care Research Group. BMJ.

[bib24] Macpherson C.C. (2004). To strengthen consensus, consult the stakeholders. Bioethics.

[bib25] Marshall P.A., Rotimi C. (2001). Ethical challenges in community-based research. American Journal of Medical Sciences.

[bib26] Mills E.J., Singh S., Singh J.A., Orbinski J.J., Warren M., Upshur R.E. (2005). Designing research in vulnerable populations: lessons from HIV prevention trials that stopped early. BMJ.

[bib27] Mitchell K., Nakamanya S., Kamali A., Whitworth J.A. (2002). Balancing rigour and acceptability: the use of HIV incidence to evaluate a community-based randomised trial in rural Uganda. Social Science & Medicine.

[bib28] Molyneux C.S., Peshu N., Marsh K. (2004). Understanding of informed consent in a low-income setting: three case studies from the Kenyan Coast. Social Science & Medicine.

[bib29] Molyneux C.S., Peshu N., Marsh K. (2005). Trust and informed consent: insights from community members on the Kenyan coast. Social Science & Medicine.

[bib30] Molyneux C.S., Wassenaar D.R., Peshu N., Marsh K. (2005). ‘Even if they ask you to stand by a tree all day, you will have to do it (laughter)…!’: community voices on the notion and practice of informed consent for biomedical research in developing countries. Social Science & Medicine.

[bib31] Morin S.F., Maiorana A., Koester K.A., Sheon N.M., Richards T.A. (2003). Community consultation in HIV prevention research: a study of community advisory boards at 6 research sites. Journal of Acquired Immune Deficiency Syndromes.

[bib32] NBAC (1999). Research involving human biological materials: ethical issues and policy guidance.

[bib33] New Economic Foundation (1999). Participation Works! 21 techniques for the 21st century.

[bib34] Newman P.A. (2006). Towards a science of community engagement. Lancet.

[bib35] Nuffield Council on Bioethics (2002). The ethics of research related to healthcare in developing countries.

[bib36] Parkin R.T. (2004). Communications with research participants and communities: foundations for best practices. Journal of Exposure Analysis and Environmental Epidemiology.

[bib37] Quinn S.C. (2004). Ethics in public health research: protecting human subjects: the role of community advisory boards. American Journal of Public Health.

[bib38] Sharp R.R., Foster M.W. (2000). Involving study populations in the review of genetic research. The Journal of Law Medicine and Ethics.

[bib39] Sharp R.R., Foster M.W. (2002). Community involvement in the ethical review of genetic research: lessons from American Indian and Alaska Native populations. Environmental Health Perspectives.

[bib40] Strauss R.P., Sengupta S., Quinn S.C., Goeppinger J., Spaulding C., Kegeles S.M. (2001). The role of community advisory boards: involving communities in the informed consent process. American Journal of Public Health.

[bib41] UNAIDS (2006). Creating efffective partnerships for HIV preventive trials.

[bib42] Weijer C. (2004). The quest for legitimacy: comment on Cox Macpherson's ‘To strengthen consensus, consult the stakeholders’. Bioethics.

[bib43] Weijer C., Emanuel E.J. (2000). Ethics. Protecting communities in biomedical research. Science.

[bib44] Weijer C., Goldsand G., Emanuel E.J. (1999). Protecting communities in research: current guidelines and limits of extrapolation. Nature Genetics.

[bib45] Weijer C., Miller P.B. (2004). Protecting communities in pharmacogenetic and pharmacogenomic research. The Pharmacogenomics Journal.

